# A Low-Noise Solid-State Nanopore Platform Based on a Highly Insulating Substrate

**DOI:** 10.1038/srep07448

**Published:** 2014-12-12

**Authors:** Min-Hyun Lee, Ashvani Kumar, Kyeong-Beom Park, Seong-Yong Cho, Hyun-Mi Kim, Min-Cheol Lim, Young-Rok Kim, Ki-Bum Kim

**Affiliations:** 1 Department of Materials Science and Engineering, Seoul National University, Seoul 151-742, Korea; 2 Institute of Life Sciences and Resources and Department of Food Science and Biotechnology, College of Life Sciences, Kyung Hee University, Yongin 446-701, Korea; 3 WCU Hybrid Materials Program, Department of Materials Science and Engineering, Seoul National University, Seoul 151-742, Korea

## Abstract

A solid-state nanopore platform with a low noise level and sufficient sensitivity to discriminate single-strand DNA (ssDNA) homopolymers of poly-A_40_ and poly-T_40_ using ionic current blockade sensing is proposed and demonstrated. The key features of this platform are (a) highly insulating dielectric substrates that are used to mitigate the effect of parasitic capacitance elements, which decrease the ionic current RMS noise level to sub-10 pA and (b) ultra-thin silicon nitride membranes with a physical thickness of 5 nm (an effective thickness of 2.4 nm estimated from the ionic current) are used to maximize the signal-to-noise ratio and the spatial depth resolution. The utilization of an ultra-thin membrane and a nanopore diameter as small as 1.5 nm allow the successful discrimination of 40 nucleotide ssDNA poly-A_40_ and poly-T_40_. Overall, we demonstrate that this platform overcomes several critical limitations of solid-state nanopores and opens the door to a wide range of applications in single-molecule-based detection and analysis.

Nanopores have attracted considerable attention because of their potential applications in the detection and analysis of single biomolecules, such as DNA, RNA, and proteins^1^,^2^,^3^,^4^,^5^,^6^. In particular, DNA sequencing has driven the technology forward due to its inherent sensitivity, high throughput, amplification-free sample preparation, and lack of labels^7^,^8^,^9^,^10^,^11^,^12^. The sub-molecular details of an individual molecule can be gathered via recording modulations in the ionic current when a molecule passes through the nanopore under the effect of a voltage applied across the pore. A series of impressive developments has been made in the area of protein nanopores using either α-hemolysin^7^,^8^,^9^,^10^ or MspA^11^,^12^,^13^ as they possess a low noise level and guarantee the reliable formation of a small pore aperture (1.5 nm) and an extremely thin sensing zone of approximately 1 nm. Combining these advantages with a recently developed method for slowing down DNA translocation using phi29 polymerase enzymes^10^,^13^ makes this platform quite promising. In contrast, the development of solid-state nanopores has been much slower and limited to merely detecting the translocation of DNA molecules, although solid-state nanopore platforms have obvious advantages over their biological counterpart such as high stability, an adjustable geometry, controllable surface properties, and the potential for integration into stand-alone devices^14^,^15^,^16^.

The reliable formation of small nanopores (< 2 nm in diameter), fabrication of an extremely thin sensing zone with a thickness comparable to the spacing of each nucleotide, decrease of the noise level, and control of the translocation speed that would guarantee sufficient time to sense each nucleotide are the few challenges that limit the performance of solid-state nanopores. Among these issues, the excess noise level in solid-state nanopores (a few tens of pA to 100 pA: ~10 times larger than that of protein counterparts^17^,^18^,^19^,^20^) has been one of the key issues responsible for the degraded signal-to-noise ratio and temporal and spatial resolution. In particular, the elevated parasitic capacitance generates a high level of dielectric noise that prevents sampling at high bandwidths. To effectively reduce the dielectric noise, several methods have been adopted such as thick dielectric layer deposition underneath an active SiN_x_ membrane^21^,^22^,^23^, PDMS sealing^22^,^24^, the utilization of glass nanopores^25^,^26^, and machining of solid-state nanopores between dielectric membranes^27^. All these platforms have resulted in a significant reduction of the noise level followed by some ramifications such as the need for additional processing to insert a thick SiO_2_ layer underneath the SiN_x_ layer and the reduction of the SiN_x_ (~80 nm) thickness to the scale of sub-10 nm. In addition, the manual coating of PDMS sacrifices accuracy and reproducibility; the fabrication of small size pores (<10 nm) is a great challenge in glass nanopores; and the utilization of PDMS as a substrate material has the disadvantage of being a non-industry standard substrate. Moreover, the involvement of too many fabrication steps may result in a trade off in the reproducibility, magnify the error level, and reduce the productivity. Therefore, a platform consisting of a low noise level and a small sensing zone length and relatively easy fabrication would offer a promising route to improving the signal-to-noise ratio for better electrical identification of single molecules.

In the present study, we propose a novel solid-state nanopore platform with a sub-10 pA noise level by fabricating a SiN_x_ membrane directly on top of highly dielectric substrates. We demonstrate that high-frequency noise signals can be significantly reduced by replacing the commonly used Si substrate with an insulating one. This platform allows easy fabrication and flexibility in selecting the thickness and material of the nanopore membrane. In spite of all these advantages, the insulating substrate faces the challenge of being a non-industry standard substrate. To enhance the spatial resolution, we utilize an extremely thin SiN_x_ membrane with a physical thickness of 5 nm and a pore size as small as 1.5 nm. Finally, we demonstrate that this device is capable of successfully detecting 40-nucleotides (nt) homopolymer single-strand DNA translocation events with a resolution of discriminating between poly-A_40_ and poly-T_40_ molecules.

## Results and Discussion

**Figure 1:**
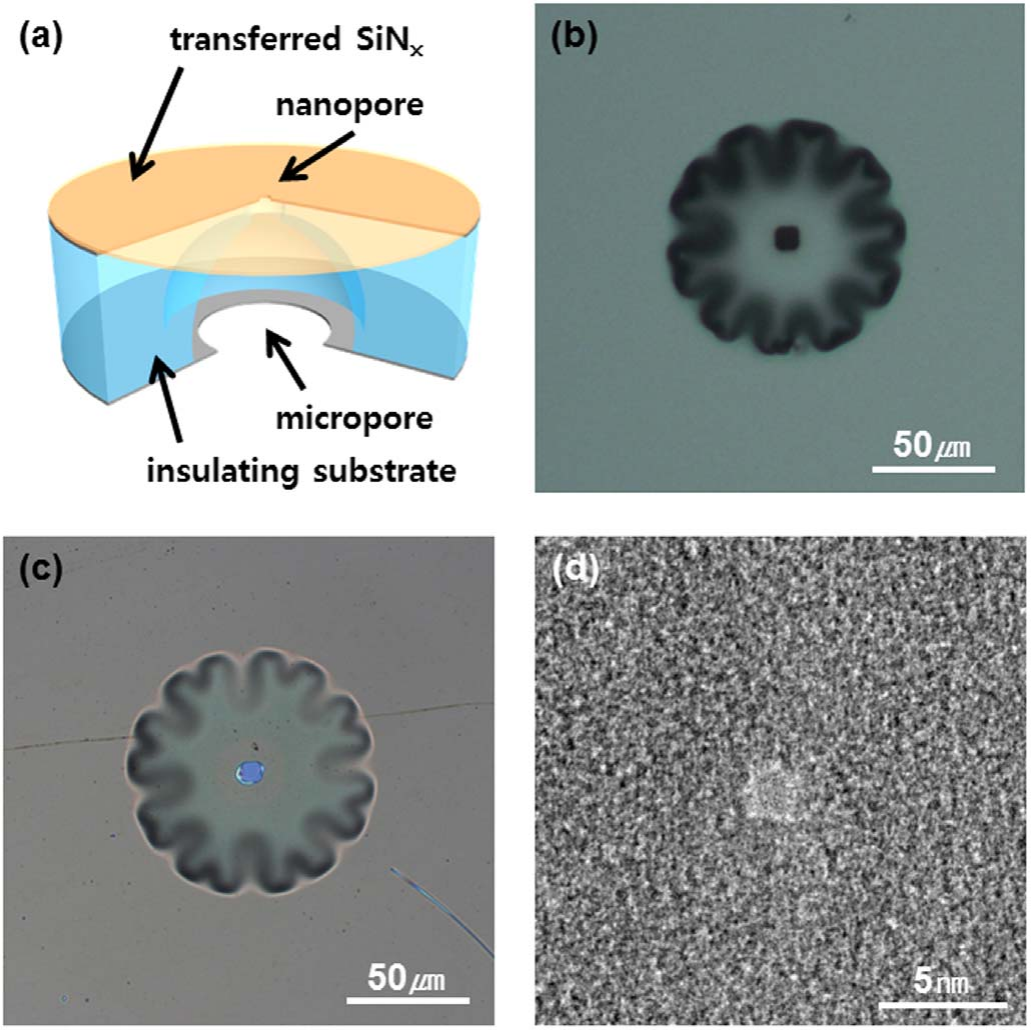
Quartz-substrate-based nanopore. (a), A schematic diagram of a quartz-substrate-based nanopore device consisting of a micrometer-sized pore in a quartz substrate and few nm-thick free-standing SiN_x_ membrane. The SiN_x_ membrane was transferred to the quartz substrate using the “fishing method”. Optical microscope image of micrometer size pore formed in quartz (b), before membrane transfer and (c), after the SiN_x_ membrane transfer. (d), TEM image of a 1.5-nm-diameter nanopore drilled by a highly focused electron beam in TEM.

Figure 1a presents a schematic diagram of the dielectric-substrate-based solid-state device. Fabrication details are provided in methods and supplementary information 1. Unlike the conventional solid-state nanopore fabrication scheme that usually employs a Si substrate due to the well-established and relatively easy fabrication of the membrane structure, we use a highly insulating quartz substrate to reduce the ionic current noise level. To define a microfluidic channel through the quartz substrate, an a-Si layer (200-nm thick) was deposited on both sides of the quartz substrate. The top layer of a-Si is opened by photolithography and reactive ion etching with an aperture size of 5 × 5 μm^2^; thereafter, the quartz substrate was slightly etched using HF (<10 μm depth). Then, the opening area (100 × 100 μm^2^) of the bottom a-Si layer was defined using the same patterning process, and a through pore was perforated using wet-chemical etching (Figure 1b). A SiN_x_ layer of 5–20 nm thickness was separately prepared as described in the methods section and transferred onto the top of this substrate using the so-called “fishing method”^28^ (Figure 1c). This method is similar to the process of transferring graphene or other 2D materials^28^,^29^. Finally, nanopores of various sizes (as small as 1.5 nm) were drilled by transmission electron microscopy (TEM) using a highly focused electron beam as illustrated in Figure 1d.

### Noise characteristics of solid-state nanopores

**Figure 2:**
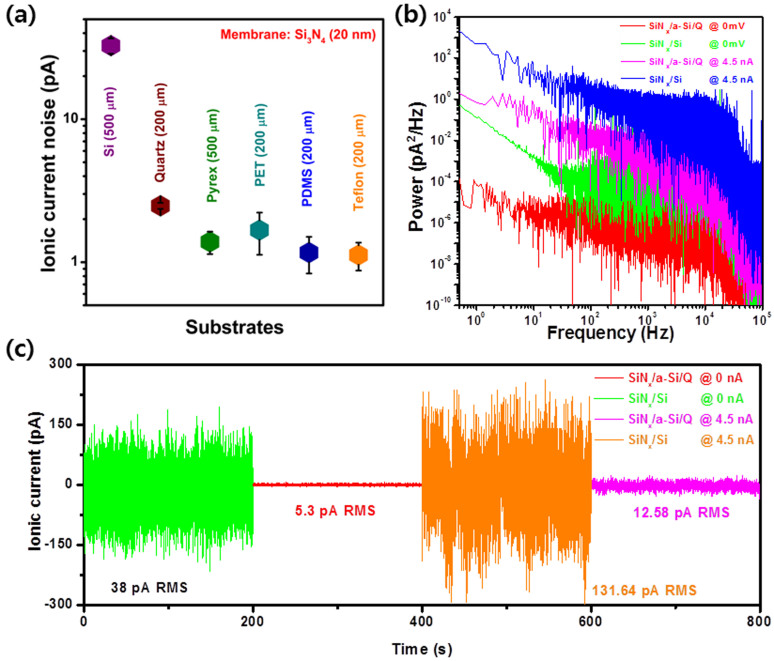
Ionic current noise in quartz-substrate-based nanopore. (a), RMS ionic current noise through 20-nm-thick SiN_x_ membrane on various substrates. (b), Power spectrum of SiN_x_ 20 nm on Si and a-Si 200 nm/quartz 200 μm measured in 1 M KCl while maintaining a pore current of 0 and 4.5 nA, respectively. (c), Baseline ionic current noise traces corresponding to the PSD curved structures described in (b).

Figure 2a shows a comparison between the ionic current noise of the nanopore devices fabricated on a Si substrate using several types of insulating substrates such as quartz, Pyrex, PET, PDMS, and Teflon. The dielectric substrate based nanopore device typically exhibits a noise level of sub-10 pA RMS, which is almost 10 times lower than the Si-based nanopore device (I_rms_~38pA). The current noise is mainly suppressed due to the high dielectric nature of the substrate, as it has been reported that materials with lower dielectric constant and dielectric loss factor (*ε* = 3.8 and *D_loss_* = ~10^−4^ for quartz, *ε* = 4.6 and *D_los_*_s_ = 3.7 × 10^−3^ for glass)^30^ provide lower ionic current noise than those composed of Si (*ε* = 11.8 and *D_los_*_s_ = 5–15 × 10^−3^)^31^. Recently, a reduced capacitive noise has been demonstrated in glass nanopores^25^,^26^ and solid-state nanopores machined in thin dielectric polymeric membranes^27^.

Figure 2b present the power spectral density (PSD) curves of Si and quartz-based nanopores measured with and without applying a voltage, which clearly depict the reduced noise spectrum of the quartz-based device in the entire frequency regime. The PSD curves of Si and quartz are compared at 0 mV while maintaining the pore current of 4.5 nA. In the low frequency regime, the quartz substrate results in a considerable reduction of the noise level irrespective of a voltage being applied. Either the mobility fluctuation of charged ions or the inhomogeneous surface charge density is assumed to be the main source of low-frequency noise at higher concentrations of KCl^32^. However, the reason for the suppression of low-frequency noise in our device is not clear. In the moderate frequency range (100–10,000 Hz), the decreased spectral density of the quartz-based device represents the suppression of dielectric noise. The noise source in this frequency range can be described as *S_D_ = 8πk_B_TD_loss_C_eff_*, where *k_B_* is Boltzmann's constant, *T* is the absolute temperature, and *D_loss_* and *C_eff_* are the loss tangent and effective capacitance of the nanopore system^23^,^33^,^34^. *‘C_eff_’* is defined as the summation of capacitances of the membrane and other parasitic elements mainly associated with the substrate and the formation of a Debye layer. The use of a high quality dielectric substrate reduces the overall effective capacitance and helps to mitigate the effect of other parasitic elements, which results in improved dielectric noise. In our devices, the membrane capacitance was measured using a cell test module using patch clamp measurements and was observed to be two orders of magnitude smaller for quartz (~70 pF) compared with Si (~1600 pF) (Supplementary Information 2). To investigate the noise characteristics in detail, the noise spectrum of the corresponding nanopore system at 0 mV was fitted to the polynomial form of *S* = *Af*^−*B*^ + *B* + *Cf* + *Df*^2^, where *f* is the frequency in Hz and *β* is the fitting parameter, which can vary from 0 to 2. The parameters *A*, *B*, *C,* and *D* represent Flicker, Johnson (Nyquist) combined with shot, dielectric and voltage noises, respectively^32^. The quartz-based device exhibits a reduced value of all the noise coefficients compared with our Si-based nanopore structure. The polynomial fit and values of all the noise parameters corresponding to the Si- and quartz-based devices are presented in Supplementary Information 3.

The noise characteristics of the Si- and quartz-based device measured at 0 mV and a pore current of 4.5 nA are presented in figure 2c, respectively. Without applying any voltage, the nanopore fabricated directly on top of the quartz substrate displayed a low noise level with a typical RMS value of 5.3 pA compared with that of the Si-based nanopore (38 pA). The observed noise level of our device is comparable to modified low-noise solid-state devices^22^,^23^,^24^,^27^,^32^. Even after applying the voltage across the pore (a different magnitude of voltage was applied for the Quartz- and Si-based devices to maintain an equal magnitude of pore current, i.e., 4.5 nA), the quartz (12.58 pA, RMS)-based device possessed superior noise characteristics compared with those of the of Si (131.6pA, RMS)-based device. It was observed that the ionic current noise level was independent of the SiN_x_ membrane thickness (varying from 5 to 20 nm) when integrated with the quartz substrate (Supplementary Information 4). The reason for this independence might be associated with the fact that the substrate's capacitance is much smaller than that of the SiN_x_ membrane, thereby dominating the effective capacitance (*C_eff_*) of the device and limiting the dielectric noise, which contributes significantly to the ionic current noise. In addition, a Si substrate does not provide a gigaohm seal in an electrolyte solution due to its relatively low resistivity (1–30 Ω-cm, B doped), while a quartz-based device exhibits a 10-times higher access resistance (Supplementary Information 2), which helps to reduce the Johnson noise. This phenomenon is clearly observed by the polynomial fit of the power spectrum as the Johnson noise coefficient of the quartz-based nanopore is two orders of magnitude lower than that of the silicon-based nanopore (Supplementary Information 3).

**Figure 3:**
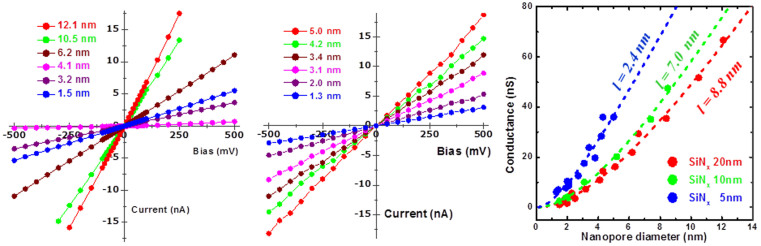
I-V characteristics of the quartz-substrate-based nanopore measured as a function of applied bias for various size pores in (a) 20-nm-thick and (b) 5-nm-thick SiNx membranes. (c) Ionic conductance versus nanopore diameter for various SiN_x_ membrane thicknesses: 20 nm (red), 10 nm (green), and 5 nm (blue). Each dotted line and the effective thickness were achieved by fitting with Equation 1, as described in the text.

Figures 3a and 3b present the I-V characteristics of quartz-based devices with nanopores of different diameters fabricated using 20 and 5 nm thick SiN_x_ membranes, respectively. The data reveals a well-behaved ohmic conduction down to a pore size of 1.5 nm even in a 5-nm-thick SiN_x_ membrane. Figure 3c plots the conductance as a function of the nanopore diameter with SiN_x_ membranes of different thicknesses, i.e., 20 nm (red), 10 nm (green), and 5 nm (blue). The variation of the conductance as a function of the diameter can be described as 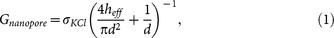
where *σ_KCl_* (11.1 *S/m*) is the molar conductivity of the electrolyte, *h_eff_* is the effective thickness of the nanopore, and *d* is the diameter of the nanopore estimated from TEM^17^,^35^. Effective thicknesses of 8.8, 7.0, and 2.4 nm were obtained for 20, 10, 5 nm thick SiN_x_ membranes, respectively. The effective thickness is smaller than the physical thickness of a transferred membrane due to the hourglass shape of the nanopore^36^,^37^. The open pore conductance (~6.95 nS, measured in 1 M KCl) of the device composed of a 5-nm-thick membrane with a 1.5 nm pore diameter was 6–7 times higher than that obtained for α-hemolysin and approximately 4 times higher than that of MspA (Table 1). Venta et al. also reported a similar trend of pore conductance in a solid-state nanopore, which was 3–14 times higher than the conductance level of protein nanopores^23^. However, the exact reason for such a high conductance level in solid-state nanopores remains unclear.

**Figure 4:**
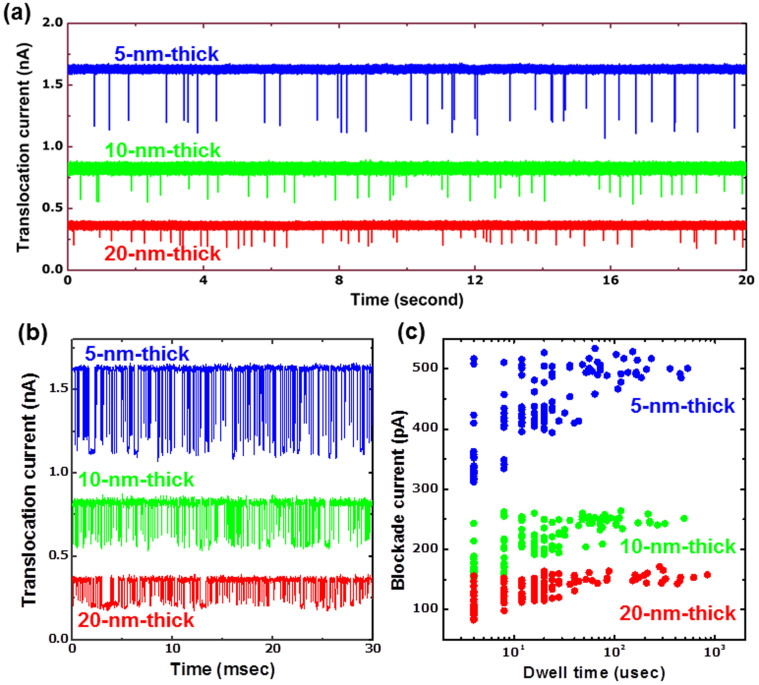
Measurement of 40 nt ssDNA using different thickness nanopores. (a), Ionic conductance as a function of time with 40 nt ssDNA through different thickness nanopores with ~2.5 nm diameter and 200 mV. The translocation signals were enhanced in thinner nanopores, which provided us with a higher signal-to-noise ratio. (b), Concatenated sets of translocation events of 40 nt ssDNA. (c), Distribution of blockade current and dwell time of > 200 events for nanopores with various thicknesses.

To investigate the signal-to-noise ratio of quartz-based solid-state nanopores, the signal characteristics derived from the translocation of 40 nt ssDNA homopolymers were analyzed. Figures 4a and 4b (concatenated plot) illustrate the ssDNA translocation event through nanopores with different membrane thicknesses and a diameter of ~2.5 nm at 200 mV. First, it is noted that the open pore current (*I*_0_) and blockade current (Δ*I_B_*) increase from 0.35, 0.81 and 1.64 nA and 0.11, 0.21 and 0.42 nA as the membrane thickness decreases to 20, 10 and 5 nm, respectively. However, the blockade fractions (Δ*I_B_*/*I*_0_) were similar regardless of the membrane thickness (29.5 ± 5.7% for 20 nm, 25.4 ± 4.5% for 10 nm, and 25.3 ± 3.3% for 5 nm thickness), suggesting that this fraction depends on the relative cross-sectional area of the DNA and nanopore rather than the membrane thickness. In contrast, the signal-to noise ratio (SNR) estimated using the formula Δ*I_B_*/Δ*I_RMS_* was observed to be 12.75 ± 2.39, 17.81 ± 3.18, and 47.50 ± 8.26 for 20, 10 and 5 nm thick SiN_x_ membranes, respectively. An analogous improvement in the SNR with decreasing membrane thickness has also been reported, with an increase in the signal-to-noise ratio from 10 to 46 as the membrane thickness decreases from 25 to 6 nm^17^. Figure 4c shows the blockade current and dwell time plot estimated from DNA translocation data. The dwell time exhibits an ample variation ranging from 4 μsec up to approximately 1000 μsec, which indicates considerable fluctuation in the translocation time. A decrease in the membrane thickness leads to a large variation in the blockade current. However, this phenomenon is not clearly understood and is definitely another intriguing subject of study. The phenomenon of a broad blockade current in thinner nanopores was previously observed and explained as being the result of the varying interactions between the DNA and the edge of the nitride membrane during translocation^38^,^39^.

**Figure 5:**
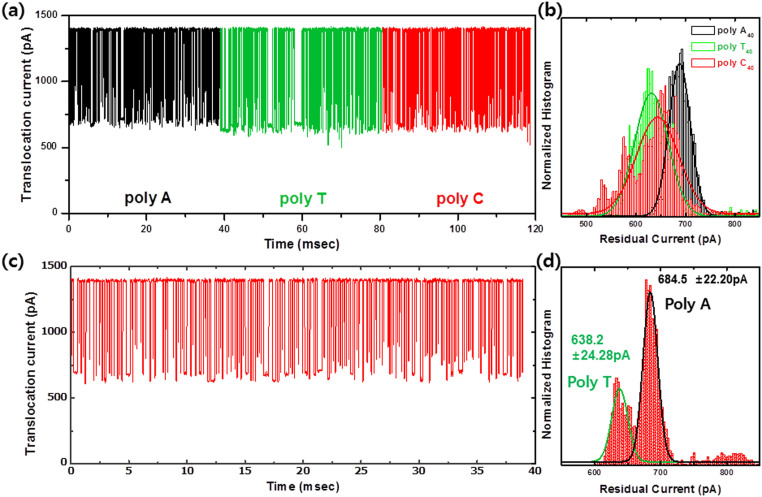
Discrimination of 4 different ssDNA homopolymers using quartz-substrate-based nanopore. (a), Concatenated sets of translocation events from each homopolymer (poly-A_40_, poly-T_40_ and poly-C_40_) using a nanopore consisting effective thickness and diameter of 2.4 and 1.4 nm, respectively at 200 mV. (b), Normalized histogram of the residual current amplitudes, which reveal the well-characterized distribution of poly-A_40_ and poly-T_40_, and a comparatively broad distribution of poly-C_40_. (c), Concatenated sets of translocation events for the mixture of poly-A_40_ and poly-T_40 _homopolymers. (d), Normalized histogram of translocation events for a mixture of the homopolymers. Poly-A_40_ and poly-T_40_ exhibited values almost identical to those obtained from individual homopolymer translocation experiment.

The membrane thickness of our solid-state device remains much larger (5 nm in physical thickness and 2.4 nm in effective thickness from electrical measurement) than the spacing of nucleotides, and the translocation speed is quite fast for identifying each nucleotide of DNA because we attempted to determine whether the 40-nt homopolymers of poly-A_40_, poly-T_40_ and poly-C_40 _could be discriminated individually. We did not observe any systematic translocation events for poly-G_40_ because it easily forms a secondary structure or G-tetrad in an electrolyte solution^7^,^40^. Figure 5a shows the multiple translocation events of ssDNA homopolymers, poly-A_40_ (black), poly-T_40_ (green) and poly-C_40_ (red) through the 1.5 nm diameter nanopore in an independent experiment. Figure 5b presents a normalized histogram of residual current. Poly-A_40_ and poly-T_40_ exhibit a definite one Gaussian peak at 688.4 ± 43.1 pA (49.5% I_0_) and 631.5 ± 67.6 pA (45.4% I_0_), respectively. Poly-C_40_ also exhibits a notable Gaussian distribution (643.8 ± 85.2 pA, 46.3% I_0_) located between poly-A_40_ and poly-T_40_. These findings were confirmed in a separate experiment using a mixture of homopolymers (Fig. 5c). The mixture of poly-A_40_ and poly-T_40_ homopolymers resulted in two clear and distinct Gaussian peaks at 679.9 ± 21.3 and 641.2 ± 20.2 pA. These results were similar to those of individual experiments and demonstrate the possibility of identifying different nucleotides of poly-A_40_ and poly-T_40_. The magnitude of the current blockade for each nucleotide (T<C<A) is also relatively well matched with the most frequently observed results, although controversy still remains (Table 1).

In conclusion, this study presents a novel process for fabricating well-defined solid-state membranes on whole quartz substrate. This platform exhibits significantly reduced noise characteristics in entire frequency regimes with sub-10 pA RMS ionic current noise. The utilization of a 5-nm-thick membrane that allows an effective pore thickness of 2.4 nm enables a greatly improved signal-to-noise ratio and spatial resolution. Additionally, this platform permits successful electrical discrimination of 40-nt ssDNA homopolymers (poly-A_40_, poly-T_40_, and poly-C_40_). The novel fabrication process we established is suitable for producing well-defined nanopore sensors that are comparable to the sophisticated protein pore on the full wafer scale. We anticipate that our technique will be further optimized for molecular diagnostic procedures and ultra-fast DNA sequencing.

## Methods

Most of the devices were fabricated on 10 × 10 mm^2^ insulating substrates. For the fabrication of the SiO_2_-containing substrates (300-μm-thick Pyrex and 200-μm-thick quartz substrates), a layer of a-Si (200-nm thick) was deposited on both sides of the substrates using LPCVD. The deposition temperature, pressure and SiH_4_ gas flow were maintained at 550°C, 250 mTorr and 60 sccm, respectively. This layer served as a masking layer during HF wet-chemical etching. For asymmetric micropore fabrication, an aperture of 5 × 5 μm^2^ was formed using photolithography and reactive ion etching (RIE) of the a-Si layer using SF_6_ gas. Wet-chemical etching was performed using 49 wt.% HF (5 min for the Pyrex substrate, 20 min for the quartz substrate). A protective film (dicing tape) was layered on the top side for mechanical stability and to provide protection during the bottom surface etching using HF. Thereafter, a 100 × 100 μm^2^ opening area was defined on the other side of the substrate by photolithography and RIE. Wet-chemical etching was performed until the bottom chamber was connected to the top chamber. The fabricated micrometer-scale pores had an asymmetric hourglass shape with a 5 μm × 5 μm opening on the top. The protective layer (dicing tape) was then removed.

An active membrane was prepared separately. Initially, a donor substrate was prepared by depositing a 500-nm-thick Ni metal film onto a Si substrate using thermal evaporation. A SiN_x_ layer with different thicknesses (5–20 nm thick) was then deposited using PECVD (13.56 MHz plasma with 1200 sccm N_2_, 800 sccm 5% SiH_4_/N_2_, and 10 sccm NH_3_ at 580 mTorr, 60 watt, 300°C). The transfer process began with spin coating of a 300-nm PMMA (950 PMMA A4, MicroChem Corp.) resist on the top of this wafer, thus creating a PMMA/SiN_x_/Ni/Si structure. The Ni film was dissolved in a FeCl_3_ solution to create a PMMA/SiN_x_ free-standing membrane floating on the solution. This membrane was carefully cleaned in deionized water and transferred onto the insulating substrate containing a micropore. The membrane was placed on the substrate and dried at room temperature. Finally, the PMMA layer was dissolved using acetone.

Finally, the nanopore was perforated using a JEOL 2010F TEM with a modified TEM holder. To adjust the pore size and shape, the current of the electron beam was controlled from 1 nA to 8 nA. The final pore size was set using a reduction method with an unfocused electron beam as previously described^41^.

The nanopores were soaked in EtOH for several minutes before the measurement. All the experiments were performed using 1 M KCl buffered at pH 8.0 with 1 mM EDTA and 10 mM Tris-HCl. The nanopore chip was installed between two buffer electrolytes with a custom-made jig composed of PDMS. In each electrolyte, a Ag/AgCl electrode connected to a patch clamp amplifier system was inserted. The ionic current measured with an Axopatch 200B amplifier was digitized at 250 kHz with a 10-kHz 6-pole Bessel filter. For the ssDNA homopolymer analysis, we used poly-A_40_, poly-T_40_, poly-C_40_, and poly-G_40_ purified using the PAGE method (Bionics Co., Ltd.) and stored at -20°C. For the study, 1 nM of the ssDNA homopolymer was mixed with the electrolyte in the *cis* chamber for measurement.

Data were collected with Clampex (MDS Analytical Tech.) and analyzed with Matlab (Mathworks). DNA translocation was identified using current thresholds greater than the noise level with baseline correction. For all the DNA samples, data were acquired for a 1.5-nm nanopore with a thickness of 5 nm. The mean current values and standard deviation values described in this report were calculated based on the peak value and standard deviation of a Gaussian fitted to the histogram of mean residual currents.

## Author Contributions

M.-H. L. and K.-B. P. conceived and designed the experiments. M.-H. L., K.-B. P. and S.-Y. C. designed, fabricated and characterized the nanopore device. M.-H. L., K.-B. P. and M.-C. L. developed and performed the nanopore experiments with ssDNA. M.-H. L., A. K. and K.-B. P. analyzed the data. M.-H. L., A. K., H.-M. K. Y.-R. K. and K.-B. K. wrote the manuscript, and all the other authors commented on the manuscript.

## Supplementary Material

Supplementary Information A Low-Noise Solid-State Nanopores Platform for Identifying Molecular Type of DNA Homopolymers

## Figures and Tables

**Table 1:** Blockade conductance of each nucleotide through a nanopore device compared with those of protein nanopores. The blockade conductance of each nucleotide through an α-hemolysin and MspA protein nanopore and open conductance of nanopore at a given (optimal) bias. The order of blockade conductance is not always consistent from one measurement tool to another because the required resolution to distinguish each nucleotide is within the range of a few tens of pS. The order of blockade conductance for each ssDNA homopolymer determined with our solid-state nanopore device is listed in the last row, which followed the tendency of other measurements using protein nanopores

Type	*ΔI_block _(A) (pS)*	*ΔI_block _(T) (pS)*	*ΔI_block _(C) (pS)*	*ΔI_block _(G) (pS)*	*ΔI_open _(pS)*	*Bias (mV)*	*Rank of ΔI_block_*	*Min. ΔI (pS)*	*Ref.*
α-hemolysin	778	805	790		958	120	T>C>A	12	3’ lead [7]
	806	799	778		958	120	A>T>C	7	4’ lead [7]
	805	808	814	802	1019	160	C>T>A>G	3	3’ lead WT [8]
	639	645	656	627	1050	160	C>T>A>G	6	3’ lead MT [8]
	139	144	111	167	300	180	G>T>A>G	5	Single nt. [9]
MspA	1442	1573	1537	1476	1806	180	T>C>G>A	34	3’ lead [11]
	1490	1627	1593	1534	1801	140	T>C>G>A	34	3’ lead [11]
	1345	1500	1612	1606	1822	180	C>G>T>A	6	3’ lead [12]
	1314	1546	1360	1263	1822	180	T>C>A>G	46	6’ lead [10]
	367	489	385	425	611	180	T>G>C>A	18	3’ lead [13]
Solid state	5100	4800	4200			1000	A>T>C		[23]
	3510	3800	3670		6950	200	T>C>A	130	This work
