# Supplementary material for: A Low-Noise Solid-State Nanopore Platform Based on a Highly Insulating Substrate
Source: Sci Rep. 2014 Dec 12;4:7448. doi: 10.1038/srep07448 (PMC4264027; doi:10.1038/srep07448)
Supplement: Supplementary Information — A Low-Noise Solid-State Nanopores Platform for Identifying Molecular Type of DNA Homopolymers [file srep07448-s1.pdf]

**SUPPORTING INFORMATION**

# A Low-Noise Solid-State Nanopore Platform Based on a Highly Insulating Substrate


Min-Hyun Lee,[†] Ashvani Kumar,[†] Kyeong-Beom Park,[†] Seong-Yong Cho,[†] Hyun-Mi Kim,[†]

Min-Cheol Lim,[‡] Young-Rok Kim,[‡] and Ki-Bum Kim[†,§,*]

[†] Department of Materials Science and Engineering, Seoul National University, Seoul 151-742, Korea

[‡]Institute of Life Sciences and Resources & Department of Food Science and Biotechnology, College of Life Sciences, Kyung Hee University, Yongin 446-701, Korea

[§] WCU Hybrid Materials Program, Department of Materials Science and Engineering, Seoul National University, Seoul 151-742, Korea

[*] To whom correspondence should be addressed. E-mail: kibum@snu.ac.kr, Telephone: 82-2-880-7095, Fax: 82-2-885-5820

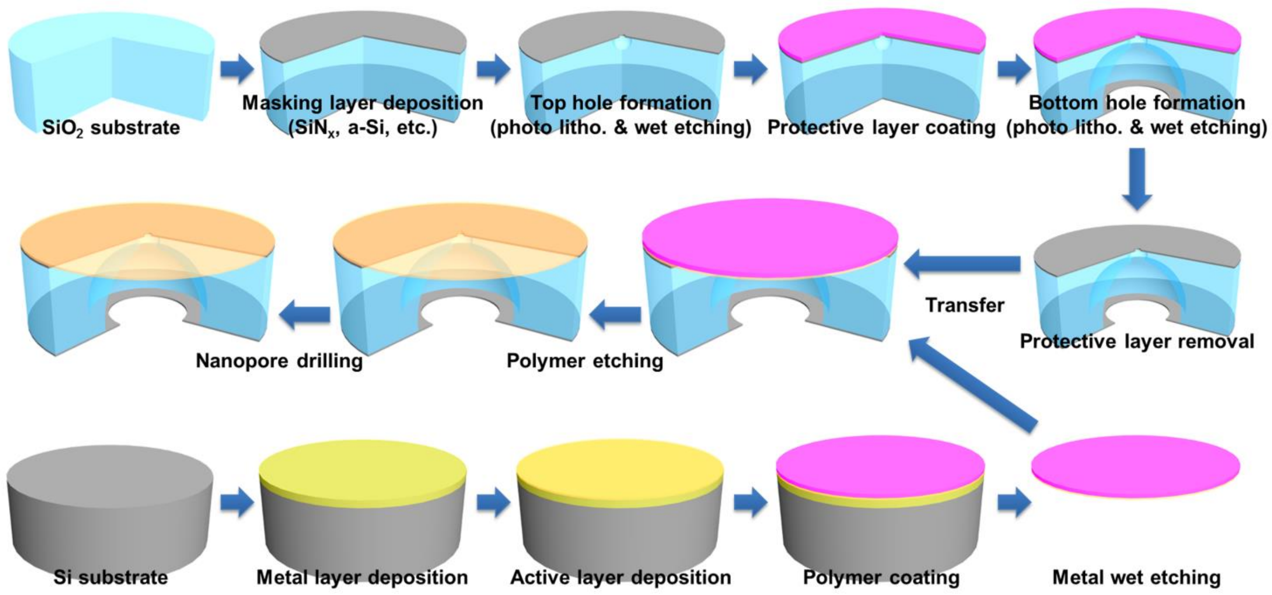


**Supplementary Information 1.** This schematic diagram illustrates the fabrication process of a dielectric substrate-based nanopore platform. An asymmetry opening through the substrate was generated by choosing different aperture size on either side of the substrate followed by photolithography and HF etching processes. It was important to create a top-surface opening as small as possible to preserve mechanical stability of the transferred $SiN_x$ membrane. In the present study, size of the top surface opening was 5x5 $\mu m^2$. The $SiN_x$ membrane layer was fabricated separately. The membrane layer was deposited on Ni (500 nm)/Si substrate and thereafter separated by wet-chemical etching of the Ni film using $FeCl_3$ solution. During the transferring process, we used PMMA as a protective layer (300-nm thick). This PMMA layer was also etched away using acetone after transfer. We referred to this process as a “fishing method” as it was a similar to one used for graphene transfer. With this technique, we can successfully transfer a $SiN_x$ layer as thin as 5 nm. Finally, the nanopore was drilled with a focused electron beam using a transmission electron microscope (TEM).

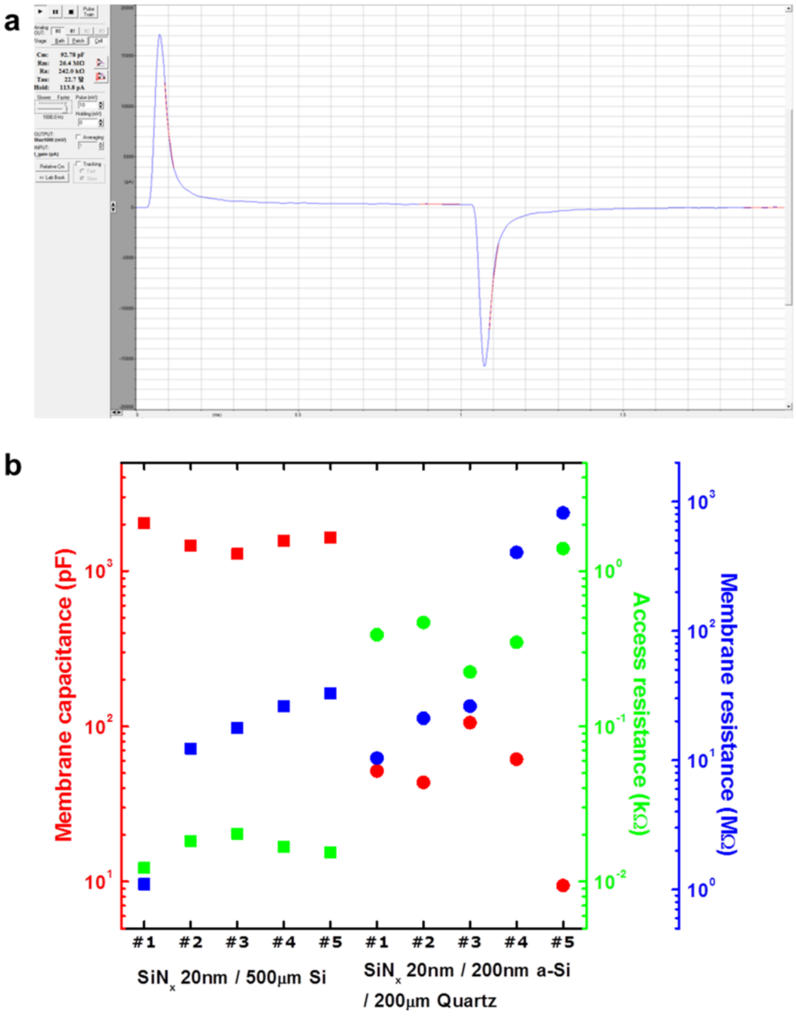


**Supplementary Information 2.** Method to calculate membrane capacitance and nanopore resistance. Membrane capacitance and nanopore resistance were measured using the “Membrane Test” mode in Clampex program (MDS Analytical Tech.) for Axopatch 200B (Figure (a)). Membrane capacitance (red), access resistance (green), and membrane resistance (blue) as a function of device type, ($SiN_x$(20nm)/Si(500μm) (square) and ($SiN_x$(20nm)/a-Si(200nm)/quartz(200μm) (circle) are shown in figure (b). Membrane resistance (blue) was related to nanopore size regardless of the device structure. The RC time constant was calculated using the product of membrane capacitance and access resistance. The quartz substrate-based nanopore had a lower membrane capacitance and higher access resistance as compared to the Si based device.

**(a)**

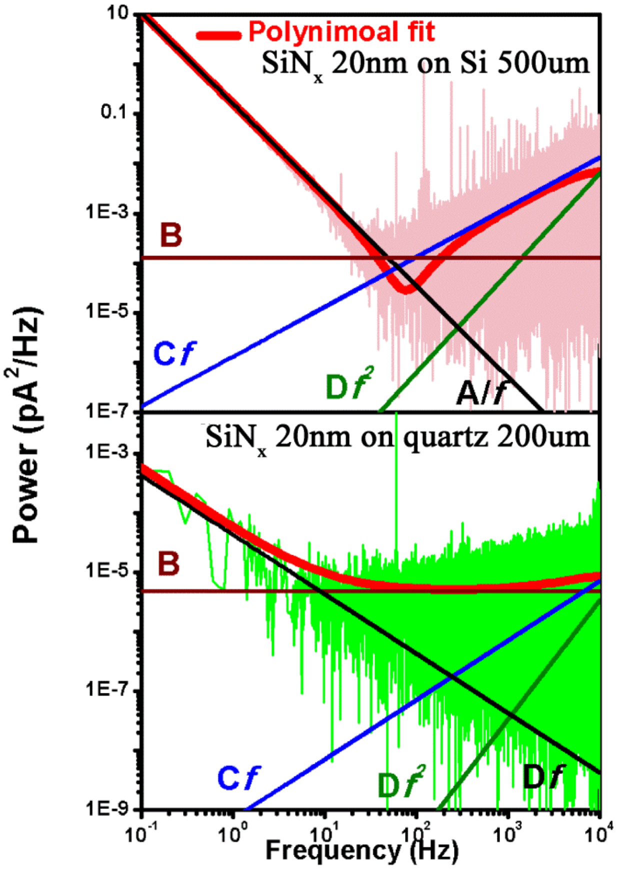


## Table

| Noise coefficients | Si based nanopore | Quartz based nanopore |
|---|---|---|
| A | $(1.66\pm0.03)\times10^{-12}$ | $(3.50\pm0.98)\times10^{-12}$ |
| B | $(1.29\pm0.62)\times10^{-4}$ | $(4.84\pm0.19)\times10^{-6}$ |
| C | $(1.33\pm0.30)\times10^{-6}$ | $(7.19\pm0.90)\times10^{-10}$ |
| D | $(6.38\pm0.28)\times10^{-11}$ | $(3.38\pm0.87)\times10^{-14}$ |
| β | 1.83±0.001 | 1.03±0.03 |

**Supplementary Information 3.** Figure 'a' shows the power spectrum of Si ($SiN_x$(20nm)/Si(500μm) and quartz ($SiN_x$(20nm)/a-Si(200nm)/quartz(200μm) based solid-state nanopore device with a polynomial fit. These power spectra were fitted using the formula $S = Af^{-\beta} + B + Cf + Df^2$, where *A*-*D* and $\beta$ are the fitting parameters. The calculated noise parameters are tabulated in above table, which suggest that all the noise components correspond to quartz based nanopore possess very low value as compared to Si based nanopore. Each type of current noise was described by equation 1-4 in the equivalent circuit with a device resistance ($R$) and effective nanopore capacitance ($C_p$) resulting from the liquid in contact with the nanopore chip.

$$\text{Voltage noise}: S_I = 16\pi^2 k_B T C_p^2 Re\left(1/Y\right) f^2,\ Y = \frac{j2\pi f C_p}{1 + j2\pi f R C_p} \quad (1)$$

$$\text{Dielectric noise}: S_I = 8\pi k_B T C_p D f \quad (2)$$

$$\text{Johnson noise + shot noise}: S_I = 4k_B T/R + 2Iq \quad (3)$$

$$\text{Flicker noise}: S_I = a/f^b \quad (4)$$

where $k_B T$ is the thermal energy, $Y$ is the admittance of the nanopore, $f$ is the frequency, $D$ is the dielectric loss constant, $I$ is the current, $q$ is the elementary charge, and $a$ and $b$ are the fitting values of the $1/f^b$ noise.

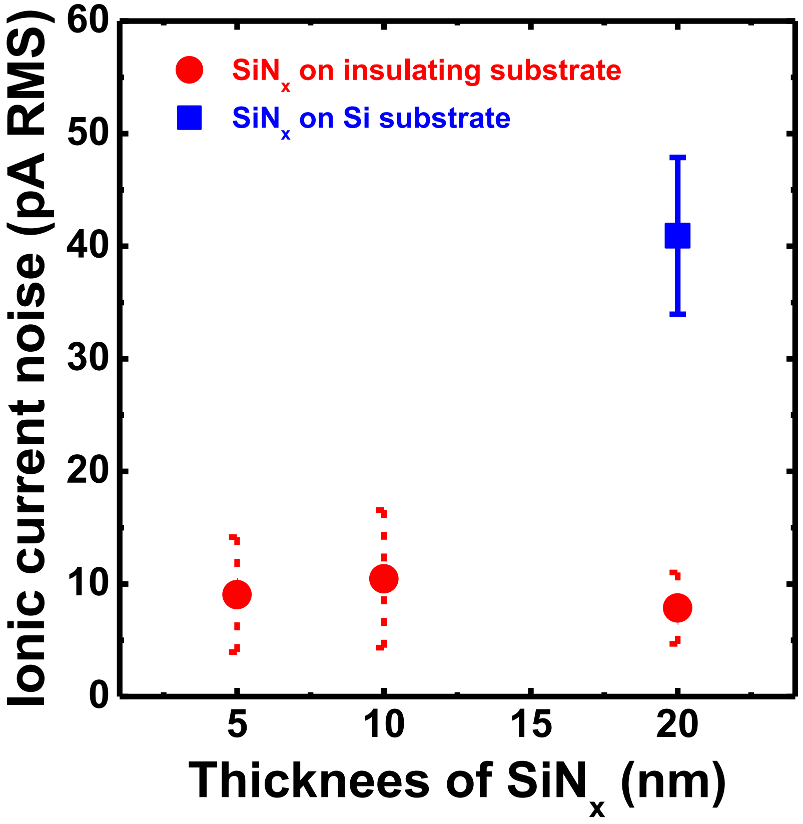


**Supplementary Information 4. Average ionic current noise as a function of SiNx membrane.** RMS noise of ionic current from the 20 nm thick SiNx on Si substrate (blue square, 11 samples), and from the 20, 10, 5 nm thick SiNx on quartz substrate (red circle, 9 samples, 5 samples, 13 samples, respectively). The RMS noise has slight related to ionic conductance of nanopore, thus, we made a statistical data with sub-10 nm diameter nanopore. The RMS noise from quartz substrate has no correlation with SiNx thickness, but, it was found to be 4 times lower than the RMS noise from Si substrate.
